# Influence of Gonadectomy on Canine Behavior

**DOI:** 10.3390/ani11020553

**Published:** 2021-02-20

**Authors:** Clara Palestrini, Silvia Michela Mazzola, Bianca Caione, Debora Groppetti, Alessandro M. Pecile, Michela Minero, Simona Cannas

**Affiliations:** Dipartimento di Medicina Veterinaria, Università degli Studi di Milano, via dell’Università 6, 26900 Lodi, Italy; clara.palestrini@unimi.it (C.P.); bianca.caione@unimi.it (B.C.); debora.groppetti@unimi.it (D.G.); alessandro.pecile@unimi.it (A.M.P.); michela.minero@unimi.it (M.M.); simona.cannas@unimi.it (S.C.)

**Keywords:** dog, gonadectomy, behavior

## Abstract

**Simple Summary:**

Veterinarians are expected to advise the owners if and when their dog’s gonadectomy should be performed and let them know if this might affect their animal’s behavior. Unfortunately, due to the lack of unequivocal scientific evidence, veterinarians differ in their opinion, with consequent diversity in advice to owners, contributing to the lack of clarity. This study aimed to evaluate the effects of gonadectomy on dog behavior across time. A total of 96 dogs, 48 gonadectomized (15 males and 33 females) and 48 entire (17 males and 31 females), were analyzed. Their owners were interviewed at time 0 (pre-surgery) and nine months later to obtain information about their dogs’ behavior. Eating behavior and dog’s weight did not show any significant changes across time in the two groups in both sexes. Gonadectomized male dogs were reported to show less mounting behavior, pull on the leash, and owner-directed aggression. Marking behavior did not vary across time for both groups of dogs. These results may contribute to shed light on which behaviors can be modified by gonadectomy over a nine-month period. However, differences in the reduction in the various types of behavior suggest that further studies of gonadectomy’s role in behavior should be conducted.

**Abstract:**

Due to the lack of unequivocal scientific evidence, gonadectomy’s effects on dogs’ behavior are still debated. Since veterinarians differ in their opinion, there may be considerable diversity in the advice received by owners. This study aimed to evaluate the effects of gonadectomy on dog behavior across time. Ninety-six dog owners (48 control dogs and 48 experimental dogs) were interviewed twice (T0 and T1, nine months later) to obtain information about their dog’s behavior. No change was found in the eating behavior or weight of dogs of both groups. Compared to T0, at T1, experimental dogs were reported to show less mounting behavior, pull on the leash, and roaming behaviors. Marking behavior did not vary across time for both groups of dogs. A tendency to reduce owner-directed aggression was observed at T1 for experimental male dogs, while no change was observed for male controls. The literature reports conflicting information about the effect of gonadectomy on behavior, suggesting that further studies about this topic should be undertaken.

## 1. Introduction

Surgical sterilization of dogs is one of the most common surgical procedures performed in veterinary practice, and it has been considered for decades to be a routine standard for the prevention of numerous undesirable behaviors, medical conditions, and diseases. Spaying and castrating of dogs may have implications not just for the dog’s health, but also for their working potential, their suitability as a pet, the control of overpopulation dynamics, and, subsequently, the numbers of unwanted dogs and strays [1,2].

Veterinarians also suggest surgical sterilization thanks to its value in preventing reproductive tract disease, including pyometra and mammary neoplasia in female cats and dogs, benign prostatic hyperplasia, perineal hernias and adenomas, prostatitis, and testicular neoplasia in male dogs [1,3,4]. Recent studies, however, reported adverse effects on dogs’ health, including an increased risk of prostate cancer and diabetes mellitus in males [5] and the risk of some form of cancer in both females [6] and males [7,8]. Moreover, gonadectomy seems to be related to an increase in joint disorders [6,9].

Gonadectomy is also a useful method to control the number of dogs in the broader population and is routinely performed in shelters, in animals as young as 6–8 weeks of age as a contraception method to help with the problems of pet overpopulation [10,11,12,13]. In an increasing number of circumstances, gonadectomy is requested to attempt to eliminate objectionable behaviors [14,15,16].

The prevalence of gonadectomy varies considerably across the globe, due to cultural differences. In some countries, surgical neutering is considered part of responsible pet ownership, and the practice is strongly encouraged by veterinarians and performed routinely [17]. In Australia, there is evidence that 85% of female dogs and 77% of male dogs are gonadectomized [18], and in the United States, dogs not intended for breeding are routinely neutered, resulting in 83% of owned dogs being spayed or neutered [19]. In several Northern European countries, surgical removal of gonads is considered mutilation and is regulated by animal welfare law: in countries such as Germany, Norway, and Sweden, routine neutering is considered unethical and must be conducted for medical reasons [17]. Therefore, in Europe, gonadectomy rates remain low: in Germany, only 43.1% of dogs are gonadectomized [20], and in the UK, just 41.1% of dogs are gonadectomized [21]. Di Nardo et al. [22] reported a spaying rate of 30% in Italy, but no other official data are available regarding the overall Italian situation.

If health issues and avoiding unplanned litters of puppies are the main reasons for spaying females, the most common reasons for orchiectomy (the surgical procedure in which one or both testicles are removed) are related to the attempt to control unwanted behaviors that cause inconvenience to their owners (i.e., roaming, mounting, abnormal urination behavior, and aggression). Several studies suggest that surgical castration of male dogs improved their behavior regarding inter-male aggression, urine marking, roaming, and mounting [14,23,24,25,26,27,28], possibly due to the reduction in testosterone [29,30]. Other studies produced contradictory findings, ranging from no behavioral changes in surgically castrated dogs to significant negative post-castration changes such as increased aggression, fearfulness, excitability, anxiety, and decreased trainability [29,31,32,33,34]. An extensive review of the literature conducted in 2010 [35] concluded that the findings obtained after surgical castration in male dogs did not demonstrate clear behavioral outcomes. Another recent literature review highlighted a significant variation between breeds and gender, with the beneficial effect of desexing stronger in females than male dogs [36].

It should be emphasized that some of these studies suffered from methodological limitations: some tended to be descriptive, while some were subjective (based on owner opinion), and they were often non-quantitative, retrospective, or based on small sample sizes and without the use of control groups [35,36]. Inconsistencies in the patient categorization of various studies make it difficult to synthesize the overall dataset [37]. The risks and benefits of gonadectomy are under constant discussion, and whether and when to neuter a dog are still in debate [37]. Despite the historical tendency to recommend gonadectomy to treat problematic behaviors [38], the effect of gender and gonadal status on behavior has not yet been cleared and defined. A conscientious owner expects to be addressed by the veterinarian on the opportunity of gonadectomy, on the times in which it would be appropriate to perform it, and on the behavioral traits that could be modified by the hormonal changes resulting from the surgical intervention. Unfortunately, there is still a broad range of opinions within the veterinary population and considerable disparity in the advice given to owners, which contribute to the lack of clarity [39]. This study aimed to evaluate the effects of gonadectomy on dogs’ behavior across time, comparing the same behaviors with a control group consisting of healthy, intact, control dogs.

## 2. Materials and Methods

### 2.1. Animals

The study was carried out on 96 dogs, 32 clinically healthy male dogs (*n* = 17 control and *n* = 15 experimental group) and 64 clinically healthy female dogs (*n* = 31 control and *n* = 33 experimental group). Dogs in the experimental group (15 males and 33 females) were recruited through the Hospital of Veterinary Medicine of the University of Milan from March 2010 to December 2016, before surgical sterilization. The control group consisted of 31 healthy intact females and 17 healthy intact males, with no signs of clinical diseases, recruited from personal contacts through the hospital during the same time frame. Animals were evaluated according to the medical history, the absence of any previous illness, and the absence of drugs or dietary supplements. General clinical examinations were performed for all dogs with recording details.

According to the Italian legislation, based on the L.D. of 4 March 2014, n.26 (GU SG n.61; 14-03-2014), which transposes the Directive 2010/63/E.U., on the protection of animals used for scientific purposes, and the E.C. Decision 29 October 2012, renewed with the protocol n.02-2016, this study did not require the formal approval of the ethical committee, since it collected data through questionnaires administered to the owners of animals conducted in the hospital for routine veterinary checks or procedures. The owners were informed in detail of the research’s purpose and signed and issued a full informed written consent.

### 2.2. Data Collection

At the end of the first examination, before the surgical sterilization for the experimental group, all dog owners were asked to complete an initial questionnaire (T0). All owners were informed of and consented to a follow-up questionnaire to further characterize their dog’s behavior nine months after the first examination (T1). The second questionnaire was then mailed or discussed by phone if there was no response by mail. The questionnaire was the same, whether it was discussed by phone or filled in by the owners. Due to the study’s length, in some cases, it was not possible to have the answers to the second questionnaire. Some owners refused to cooperate, and in some cases, dogs were euthanatized (*n* = 1) or died of disease (*n* = 2) before the end of the study. At the end of the nine months, 96 owners replied, out of the 156 to which questionnaires were sent (61.53% response rate). The questionnaire included the dog’s demographic information, behavioral history, and information on its physical and social environment. Through open questions, owners provided information about the signalment and the medical and behavioral history of their dog. The other sections of the questionnaire were composed of multiple-choice questions: the first part provided information about the home environment, age of the animal (current age and age at acquisition), the origin of the dog (breeder, pet store, shelter, rescue, family, friends, or stray), and the number of other pets in the household. The questionnaire also provided information about a specific dog’s behavioral patterns (drinking, grooming, sleeping, eating, exploration, and play) and whether the dog had displayed specific behaviors compatible with management or behavioral problems (see Table 1). 

### 2.3. Surgical Procedures

After being deemed healthy, as apparent from physical and hematological analysis, dogs belonging to the experimental group underwent neutering. Bilateral pre-scrotal orchiectomy and ventral midline ovariohysterectomy were performed following standard anesthetic and surgical procedures [2,40,41]. The same surgeons performed all the neutering.

### 2.4. Statistical Analysis

Answers to the questionnaire were scored, and data were entered into Microsoft Excel (Microsoft Corporation 2010, Microsoft Italia, Milan, Italy) and analyzed with the SPPS statistical package (IBM SPSS Statistic 21,IBM Italia, Milan, Italy ). Descriptive statistics (relative proportions, minimum and maximum values, median, mean and standard deviations) were calculated to provide a general description of the two experimental groups. Data were tested for normality, and a matched-pairs Wilcoxon test was used to investigate potential differences in dogs’ behavior between time periods. Any differences in behavior, management, and environment between the two groups were assessed using a Mann–Whitney test. A logistic regression using age of adoption, age at time 0, and source of adoption as covariates was used to identify any influences on the model. Differences were considered to be statistically significant if *p* ≤ 0.05.

## 3. Results

### 3.1. Male

#### 3.1.1. Characteristics of the Sample at Time 0

A total of 15 dogs underwent gonadectomy (experimental group), and 17 entire dogs were enrolled as the control group. Age and weight are reported in percentages in Table 2 and Table 3.

Mixed-breed dogs were the most represented in both groups, even if higher percentages were reported in the experimental group (experimental, 53.3% of total; control, 23.5% of total). Pure breed dogs were equally distributed, in smaller percentages, in both groups. Dogs in our sample were adopted between two and three months of age (33.3% experimental and 58.8% control) and between three and six months (40% experimental and 17.6% control). A smaller percentage of dogs were adopted before 50 days (13.3% experimental and 11.8% controls). Only dogs belonging to the control groups (11.8%) were adopted between seven and 12 months, and only experimental group dogs (13.3%) were adopted after one year.

Most of the dogs of the experimental group were adopted from shelters (53.3%), while a smaller percentage were from private people (20%) and breeders (13.3%). On the contrary, most of the control group dogs were adopted privately (52.9%) and from breeders (35.3%), with a lower number of stray dogs from shelters (11.8%). Dogs in the experimental group lived in a rural environment (46.7%) or urban apartments (40%). Conversely, the control group dogs lived mostly in a rural environment (64.7%). No statistical differences were found in these demographic variables (Z ≤ 1.815). The experimental dogs were mostly the only animal in the household (53.3%), while 70.6% of the control dogs lived with other animals (Z = −2.342; *p* ≤ 0.05).

At T0, only the pull on the leash behavior was significantly higher in the experimental group than in the control group (80% experimental vs. 41.2% control) (Z = −2.1964; *p* ≤ 0.05). Sleeping behavior showed a tendency to be higher in the experimental dogs (13.3%) than in controls (0%) (Z = 1.904; *p* = 0.057). Behaviors such as barking (40% experimental vs. 17.6% control), roaming (46.6% experimental vs. 17.6% control), restlessness (33.3% experimental vs. 17.6% control), aggression towards owner (53.4% experimental vs. 23.5% control), aggression towards stranger (40% experimental vs. 17.6% control), inter-dog aggression (26.7% experimental vs. 11.8% control), inappropriate elimination (33.3% experimental vs. 5.9% control), and mounting (66.7% experimental vs. 58.8% control) were higher, even if not significantly, in the experimental group than in the control group (Z < 1.000).

No differences in the two groups were seen for the other behaviors considered.

We analyzed the model with logistic regression using age of adoption, age at time 0, and source of adoption as covariates, and we found an influence of age just on male dogs’ excessive greeting behavior (*p* ≤ 0.05).

#### 3.1.2. Change across Time and Differences between Groups

Answers to the questionnaires revealed, in both groups of dogs, no changes from T0 to T1 for the following behaviors: eating and drinking, sleeping, exploration, grooming, chewing objects, destruction, play, pica, coprophagia, repetitive behaviors, thunderstorm and noise phobia, and aggression towards strangers. No difference was found in the pull on the leash behavior between control and experimental dogs at T1. The weight of dogs did not show any significant changes across time in the two groups.

Owner-directed aggression did not show statistical differences between groups at T0 but showed a tendency to decrease (from 53.3% at T0 to 26.7% at T1) in the experimental group (*p* = 0.059), while in control dogs, it did not change across time.

**Figure 1:**
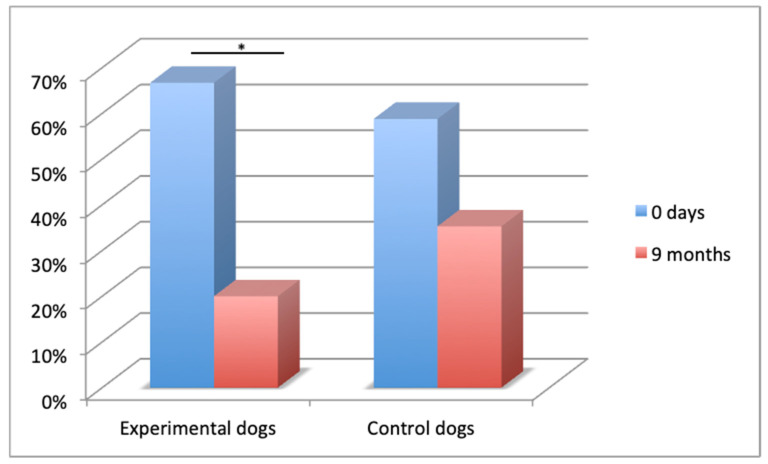
Mounting behavior in the two groups of males across time (* *p* ≤ 0.05).

Mounting behavior significantly decreased across time in the experimental group from 66.7% to 20% (Z = −2.310; *p* ≤ 0.05) (Figure 1), and no statistically significant changes were observed across time in the control group (58.8% to 35.3%).

No statistical differences between the two groups of dogs were observed at T9 for the other analyzed behaviors. Changes in behaviors are reported in Table 4.

### 3.2. Female

#### 3.2.1. Characteristics of the Sample at Time 0

A total of 33 females underwent gonadectomy (experimental group), and 31 entire females were enrolled as the control group. Age and weight are reported in percentages in Table 5 and Table 6.

Mixed-breed dogs were more represented in both groups, even if higher percentages were reported in the experimental group (experimental, 33.3% of total; control, 19.4% of total). Pure breed dogs were equally distributed, in smaller percentages, in both groups. Dogs in our sample were adopted between two and three months of age (48.5% experimental and 54.8% control) and between three and six months (27.3% experimental and 17.6% control). A smaller percentage of dogs were adopted before 50 days (21.2% experimental and 12.9% control). Instead, only dogs belonging to the control groups (6.5%) were adopted between seven and 12 months, and only experimental group dogs (3%) were adopted after one year.

Most of the experimental group dogs were adopted privately (39.4%), while a smaller percentage were from breeders (27.3) and shelters (15.2%). On the contrary, most of the control group dogs were adopted from breeders (41.9%) and privately (38.7%). Most experimental group dogs were the only animal in the household (54.5%), while the subjects in the control group lived mostly with another dog (54.8%). No statistical differences were found in these demographic variables (Z ≤ 1.676). Most experimental dogs (54.4%) lived in an urban apartment, while 54.8% of the control group lived in a rural environment (Z = −4.055; *p* ≤ 0.05).

At T0, behaviors such as restlessness (21.2% experimental vs. 6.5% control) and barking (27.3% experimental vs. 9.7% control) were higher, even not statistically significant, in the experimental group than in controls (Z ≤ 1.788). No differences in the two groups were seen for the other analyzed behaviors.

#### 3.2.2. Change across Time and Differences between Groups

Answers to the questionnaires revealed no changes across time (from T0 to T1) in both groups of dogs for the following behaviors: eating and drinking, exploration, grooming, chewing objects, destruction, play, pica, coprophagia, roaming, greeting, pull on the leash, inappropriate elimination, thunderstorm and noise phobia, and aggression. The weight of dogs did not show any significant changes over time in the two groups.

In the experimental group dogs, barking and restlessness decreased over time, even if not significantly, from 27.3% to 18.2% and from 21.2% to 15.1%, respectively. No changes were observed in the barking and restlessness of control dogs. Other behaviors decreased, even if not significantly, across time in the experimental group, and no changes were seen in the control group: mounting (from 37.4% to 21.2), repetitive behaviors (from 21.2% to 12.1%), and sleeping (from 15.2% to 3%).

No statistical differences between the two groups were observed at T1 for all the analyzed behaviors.

## 4. Discussion

The purpose of this study was to evaluate the effects of gonadectomy on dogs’ behavior across time, comparing the same set of behaviors with a control group consisting of intact, healthy dogs. There are different papers on this topic in the literature, but the effect of gender and gonadal status on behavior has not yet been fully elucidated and clearly understood. In the literature, several authors already investigated the behavioral effects of gonadectomy by interviews, but the methodology applied in their studies suggests caution in interpreting the results. Some interviews took place too long after the gonadectomy (between two and eight years), and others were conducted non-randomly [14,15,42]. To prevent these errors in the present investigation, the authors used a reliable interview form, a randomly enrolled population, and a time limit of nine months post-orchiectomy and after the last interview. A six-month period was suggested by Hopkins et al. [14] as the minimum to allow behavioral changes to develop.

Mixed-breed dogs, of both genders, were the most represented in the experimental and control groups, with higher percentages in the experimental group. This finding could be related to the fact that pure breed dogs are often kept also for possible breeding purposes, especially if they were adopted from breeders. Additionally, female mixed-breed dogs, not intended for breeding, are routinely neutered. Likewise, pure breed dogs of both genders were equally distributed, in both experimental and control groups. Although not statistically significant, a difference in the two groups’ living environment was found, with experimental dogs living mainly in an urban setting apartment, and control dogs living in a rural environment. Since there would be different behavioral expectations in rural vs. urban environments, this could be a limitation in interpreting our results and could help future research balance groups also according to the living environment.

Most of the dogs enrolled were aged between one and five years, and also the weights of dogs were similarly distributed in the two groups. In males, at T0, only the pull on the leash behavior was significantly higher in the experimental group than in the control group, and no difference between groups for this behavior was observed at T1. On the contrary, in females, no statistical differences were found in the two groups for all the analyzed behaviors. A limitation in the comparison between groups at T1 is represented by the significant difference between groups in the pull on the leash behavior at baseline. This finding may be explained by the owners’ choice to desex their dogs, hoping that some agitation behavior will be reduced.

For the owners, one of the main concerns about neutering a dog is the perspective of its overweight. In our sample, no body weight or eating behavioral changes were found in both genders and groups of dogs. In accordance with our study, Fazio et al. [43] found no significant body weight increases in ovariohysterectomized bitches two months after the intervention, but other authors reported that castrated dogs are more often obese than intact dogs [15,24,27]. There is conflicting information regarding this topic in the literature since obesity is not uniquely reported as a consequence of gonadectomy [4]. In a recent review, Urfer and Kaeberlein [36,37,38,39,40,41,42,43,44] reported that there is consistent evidence that desexing is associated with an increased risk of obesity in dogs of both genders and that sex steroids induce a decrease in caloric intake, at least in female dogs. Edney and Smith [44,45] suggested that after orchiectomy, food intake remained unchanged while energy consumption decreased, whereas Hopkins et al. [14] and Hart [24] assumed that an orchiectomy reduction in activity is related to a decline in roaming behavior. Heidenberger and Unsheim [15] also observed a post-orchiectomy decrease in activity, but they suggested that this was due to an increase in body weight, not vice versa. Reduced activity was also observed in our study. Mounting behavior decreased significantly over time in the male experimental group and a tendency to decrease was also observed in the females. Barking, repetitive behavior, sleeping, restlessness (in females), and pulling on leash (in males) decreased, even if not significantly, in the gonadectomized dogs between time 0 and 9 months, probably as a result of the decrease in the gonadal steroid hormones and thereby a reduction in sexually dimorphic behavior [14,15,24,27,28,36,37,38,39,40,41,42,43,44].

A series of studies published primarily between the 1970s and 1990s suggested that surgical castration of male dogs improved their behavior regarding roaming, mounting, and urine marking [14,23,24,25,26,27,28,31,45,46], possibly due to the reduction in testosterone [29,30]. These results confirm our findings on mounting behavior that, in the experimental group, showed a significant decrease across time: nine months after gonadectomy, it was significantly lower in gonadectomized dogs than in controls, more markedly in males.

In our sample of dogs, we did not find any change regarding inappropriate elimination after gonadectomy. Several studies described a marked decrease in urine marking after gonadectomy [1,27,28,29,37,46,47,48], especially in male dogs, and this effect was not related to the age of desexing [36,37,38,39,40,41,42,43,44]. This observation has been related to the strength of the olfactory stimuli that may evoke the scent-marking behavior, which is influenced by blood testosterone levels: a decrease in blood testosterone concentration will necessitate more potent olfactory stimuli to evoke the behavior. An absence of these stimuli inside the house may explain the decrease in scent-marking behavior observed post-orchiectomy [14,15]. However, inappropriate elimination can have different heterogeneous causes, such as inappropriate or incomplete housetraining, or can be motivated by anxiety or stressful situations [48,49,50,51].

There is a common belief that castration reduces aggression, but there is an absence of agreement in the literature. Several studies suggest that surgical castration is effective on dogs’ inter-male aggression [14,25,51,52], possibly due to the reduction in gonadal steroid hormones [29,30]. We did not observe a decrease in inter-male aggression, after nine months, in both castrated and entire male dogs, but we found a tendency to decrease in owner-directed aggression in male experimental dogs nine months after castration. On this theme, a large number of studies produced contradictory findings, including no behavioral changes in sterilized dogs or significant negative post-castration changes such as increased aggression, fearfulness, excitability, anxiety, and decreased trainability [29,31,32,33,52,53]. No significant relationship between aggressive behaviors towards familiar people or other dogs and gonadectomy was presented by Farhoody et al. [34,54].

An extensive review of the related literature conducted in 2010 [35] concluded that the data collected do not demonstrate clear behavioral outcomes following surgical castration in male dogs. More recently, Urfer and Kaeberlein [36] highlighted that the evidence for an influence of desexing on boldness-related and aggressive behavior is inconsistent and sometimes contradictory. It should also be considered that owners are often unable to distinguish the different types of aggressive behavior (i.e., competitive, pain-induced, territorial, fear-induced, predatory, maternal, and learned aggression), and that the classification of aggression varies in the different studies on this topic. At present, dog aggression causes are not sufficiently well understood to allow an accurate prediction of the effect of gonadectomy on it. Dogs’ aggressive behavior is not related to a single factor; instead, multiple environmental and genetic factors may contribute to its expression.

Another critical element to deepen is the age at which the dogs underwent gonadectomy: McGreevy et al. [38] reported that surgery timing could influence the dog’s tendency to show numerous behaviors (primarily related to fearfulness and aggression). In the literature, it is reported that gonadectomized dogs of both sexes are more fearful [38] and, in particular, Balogh et al. [53,54,55] stated that the gonadectomized female dogs were more fearful in response to loud noises. Houlidan [37] found an association with the age of gonadectomy and noise phobia, reporting that dogs gonadectomized before 5.5 months of age were more likely to display noise phobia. Similar results were reported in Spain [54,56]. On the contrary, in our study, we did not find a variation in noise sensitivity in gonadectomized dogs. Our data cannot be compared since our dogs underwent surgical intervention at different ages (the majority of dogs were gonadectomized between 1 and 5 years).

Early life experience impacts dogs’ behavior [55,56,57], and this could have a greater influence than neutering on behavioral changes. In our results, the logistic regression test, using age and source of adoption as covariates, found the influence of age just on excessive greetings only in male dogs, but this aspect could be considered when interpreting the results. For future studies, it is advisable to balance the groups by age to exclude age’s influence on behavioral changes.

## 5. Conclusions

Some aspects of the effects induced by dogs’ gonadectomy are still controversial, probably because the intervention confers a mixture of benefits and adverse effects that also depend upon the age at neutering, sex, and breed. Many veterinarians and various experts assert that companion animals not intended for breeding should be spayed or neutered. However, the decision must be made on a case-by-case basis taking into account age, breed, sex, intended use, household environment, and the dog’s temperament [37]. The single patient’s evaluation should consider the risks and benefits of gonadectomy, including potential effects on health, behavior, longevity, and the risks of anesthetic and surgical complications [37]. This study’s results may contribute to shed light on which behaviors can be modified by gonadectomy over a nine-month period, a timeframe that allows behavioral changes to develop. Unfortunately, there are still areas in which agreement between authors is scarce and where conflicting information is given to the reader. The differences in the reduction in the various types of behavior suggest that further studies of gonadectomy’s impact on behavior should be conducted.

## Figures and Tables

**Table 1:** Behaviors analyzed during the study. Variables marked with “*” were collected through a Likert scale. For Grooming, Sleeping, Exploration, and Drinking behaviors, the Likert scale had 3 points: poor, normal, excessive; instead, for the Play behavior, the Likert scale had 5 points: absent, poor, normal, strong, obsessive), and the others behaviors were collected in a binary way (Yes/No).

Analyzed Behaviors	
Mounting	Aggression towards stranger
Barking	Aggression towards owner
Eating	Inter-dog aggression
Greetings	Chewing object
Grooming *	Destruction
Sleeping *	Inappropriate Elimination
Exploration *	Pica
Drinking *	Coprophagia
Play *	Repetitive behaviors
Roaming	Restlessness
Pull on the leash	Thunderstorm and noise phobia

**Table 2:** Age of male dogs (experimental and control groups).

Age	0–6 Months	7–12 Months	1–5 Years	6–10 Years	>10 Years
Experimental	0.0%	20%	33.3%	13.3%	33.3%
Control	5.9%	11.8%	58.8%	23.5%	0.0%

**Table 3:** Weight of male dogs (experimental and control groups).

Weight	<10 kg	11–20 kg	21–35 kg	>35 kg
Experimental	20%	20%	46.7%	13.3%
Control	11.8%	29.4%	29.4%	29.4%

**Table 4:** Behaviors that changed across time between groups. * Owner-directed aggression showed a tendency to decrease in the experimental group (Z= −1.890; *p* = 0.059).

Behaviors	Experimental/Control	T0 (Baseline-Pre-Surgery)	T1 (9 Months Follow-Up)
Barking	Experimental	40%	33.3%
	Control	17.6%	17.6%
Roaming	Experimental	46.6%	33.3%
	Control	17.6%	5.9%
Inappropriate elimination	Experimental	33.3%	13.3%
	Control	5.9%	0%
Restlessness	Experimental	33.3%	20%
	Control	17.6%	11.8%
Pull on the leash	Experimental	80%	66.6%
	Control	41.2%	47.1%
Greetings	Experimental	40%	33.3%
	Control	35.3%	35.3%
Owner-directed aggression	Experimental	53.3% *	26.7%*
	Control	23.5%	23.5%
Inter-dog aggression	Experimental	26.7%	20%
	Control	11.8%	5.9%

**Table 5:** Age of female dogs (experimental and control) at T0.

Age	0–6 Months	7–12 Months	1–5 Years	6–10 Years	>10 Years
Experimental	6.1%	21.2%	39.4%	24.2%	9.1%
Control	9.7%	6.5%	51.6%	25.8%	6.5%

**Table 6:** Weight of female dogs (experimental and control) at T0.

Weight	<10 kg	11–20 kg	21–35 kg	>35 kg
Experimental	36.4%	30.3%	21.2%	12.1%
Control	38.7%	16.1.4%	45.2%	0%

## Data Availability

Please refer to suggested Data Availability Statements in section “MDPI Research Data Policies” at https://www.mdpi.com/journal/animals/instructions#suppmaterials.
